# Supplementary material for: About bacteriophage tail terminator and tail completion proteins: structure of the proximal extremity of siphophage T5 tail
Source: J Virol. 2024 Dec 23;99(1):e01376-24. doi: 10.1128/jvi.01376-24 (PMC11784142; doi:10.1128/jvi.01376-24)
Supplement: Supplemental material — Figures S1 to S3; Tables S1 and S2. [file jvi.01376-24-s0002.pdf]

About bacteriophage tail terminator and tail completion proteins: structure of the proximal extremity of siphophage T5 tail

Romain Linares[1,2] and Cécile Breyton[1]*

[1]Univ. Grenoble Alpes, CNRS, CEA, IBS, F-38000, Grenoble, France.

[2]Current address: European Molecular Biology Laboratory, Grenoble, France

*Corresponding author: Dr. Cécile Breyton: Tel: +33 4 57 42 86 31; E-mail: Cecile.Breyton@ibs.fr

Supplementary figures 1-3 and tables 1-2 (below)

PDB file of the flexible-fitted TCP_{p143} AF2 predicted model in a separate file (Supp-p143-FlexibleFit.pdb).

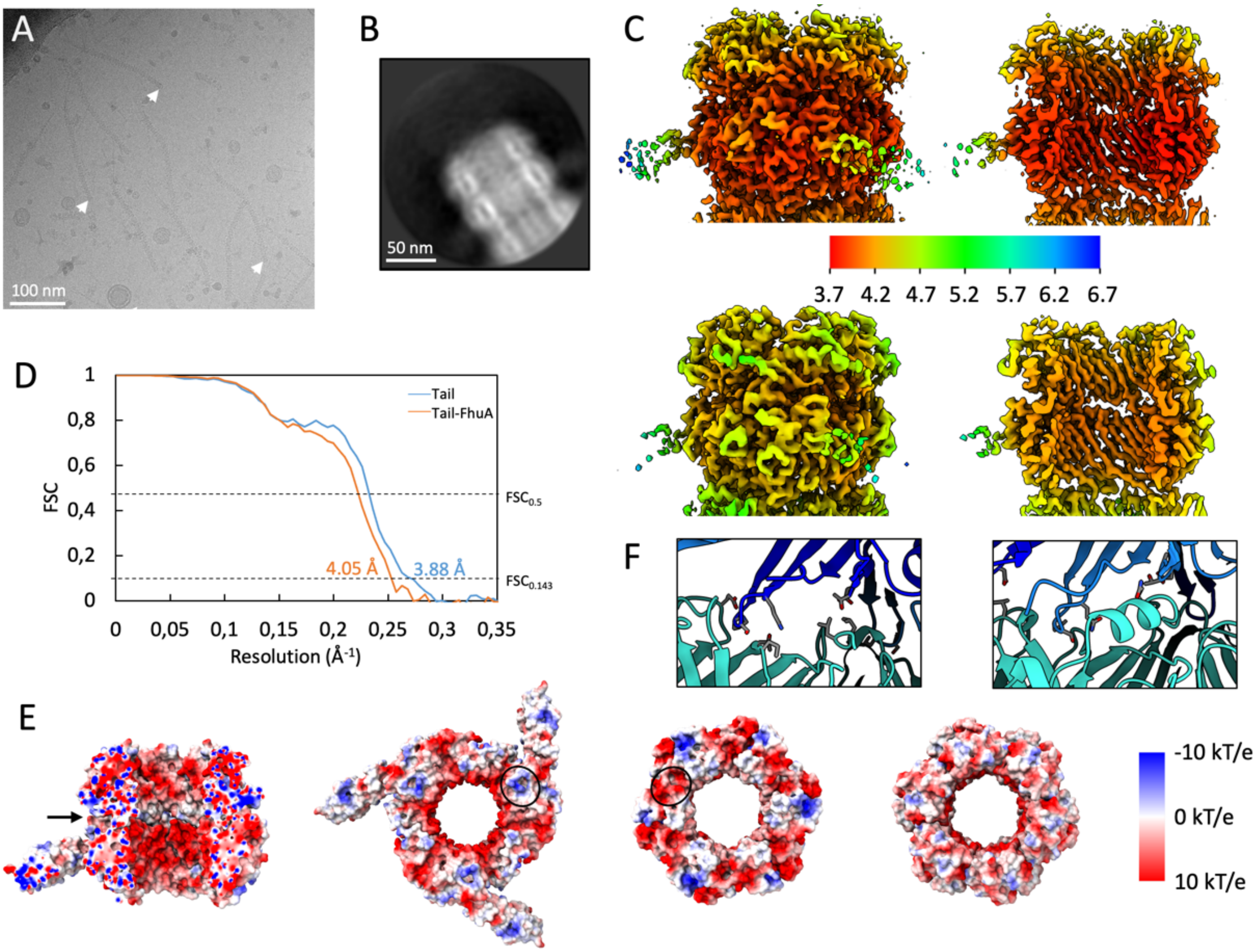

Figure S1: A. Cryo-EM micrograph of T5 tails. The Trp_{p142} hexamer forms the last ring of the proximal extremity of T5 tail and is indicated with a white arrowhead. **B.** 2D class of T5 tail proximal extremity, side view. **C.** Local resolution of the cryo-EM maps for T5 tail proximal extremities, as determined by Relion, in the tail native state (top) and after interaction with *E. coli* receptor FhuA (bottom). The left panel shows a side view for the two maps while the right panel presents an interior side view. All maps were calculated with a C3 symmetry imposed and the key is the same for both maps. The top of the Trp_{p142} hexamer appears a bit less resolved, probably because of some flexibility of the binding interface to the Head Completion Protein of the capsid (absent here). **D.** Fourier shell correlation (FSC) plot for the maps presented in C. $FSC_{0.5}$ and $FSC_{0.143}$ cutoffs are indicated, as well as the estimated resolution ($FSC_{0.143}$) for each map. **E.** Electrostatic charge distribution of the proximal extremity of T5 tail tube. From left to right: slice from a side view showing the tube lumen (panel 1), top view of the last TTP_{pb6} ring (interacting with TrP_{p142} ring, panel 2), bottom view of TrP_{p142} ring (interacting with TTP_{pb6}, panel 3) and top view of the p142 ring (directed toward the capsid, panel 4). Complementary charge patches are circled in black. The position of the TTP_{pb6} – TrP_{p142} interface is indicated with a black arrow in the first panel. Electrostatic charge distribution was calculated with the "coulombic" tool from ChimeraX. **F.** Close-up of the TrP_{p142}-TTP_{pb6} interface for TrP_{p142} monomer 1 (left, dark blue) and 2 (right, light blue). TTP_{pb6} is displayed in cyan.

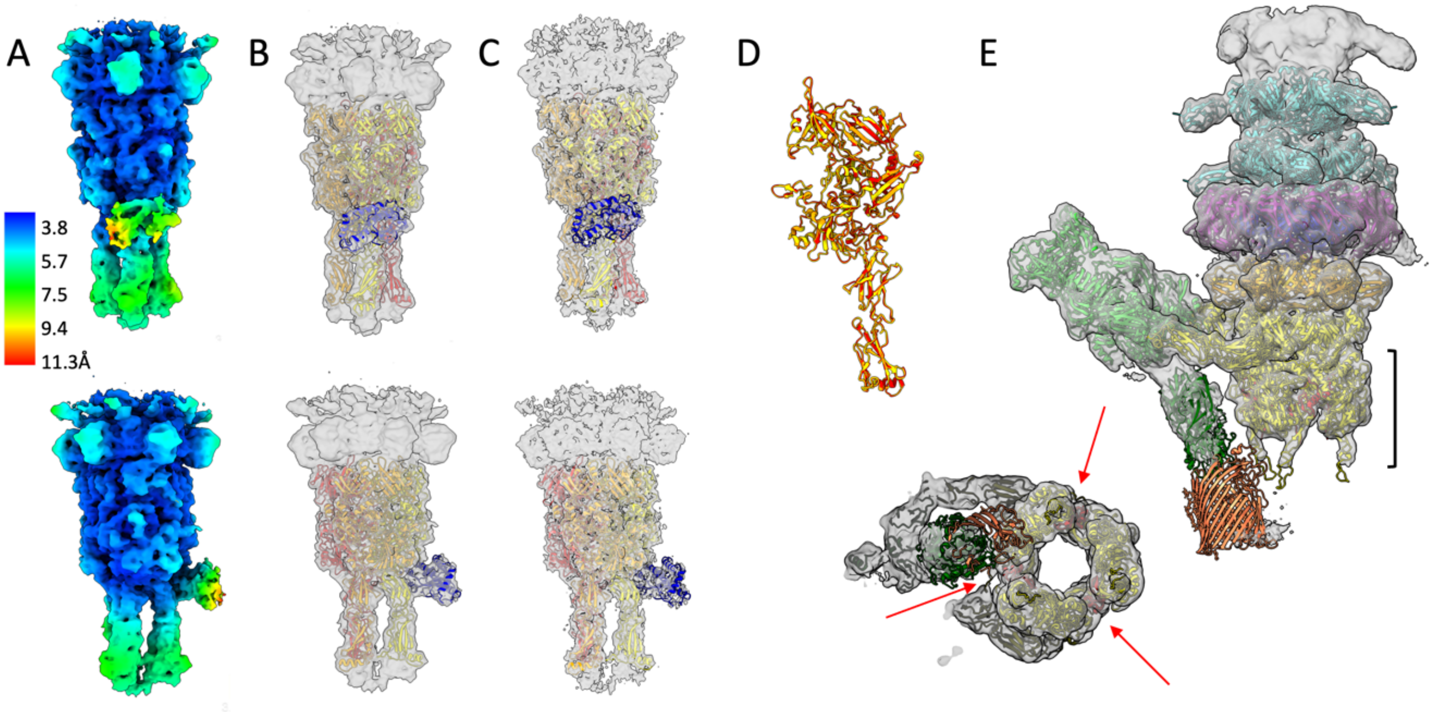

Figure S2: A. Local resolution of the unsymmetrised cryo-EM map for T5 tip as determined by Relion, front (top) and side (bottom) views. **B-C.** Unsharpened (B) and sharpened (C) unsymmetrised EM maps of T5 tip, fitted with three monomers of BHP_{pb3} (yellow, orange, red) and the monomeric TCP_{p143} (blue). **D.** Superimposition of the three BHP_{pb3} monomers, individually refined in the unsymmetrised sharpened map of T5 tip shown in C. rmsd between monomer 1 and 2 or 1 and 3 is respectively 0.368 Å and 0.353 Å over all 949 residues. **E.** EM map and model of T5 tail tip complex after its interaction with the bacterial receptor FhuA inserted in nanodisc (described in (10)). The location and thickness of the bottom slice is indicated on the first panel by a black accolade. Arrows are pointing to TCP_{p143} density in the map before interaction with the receptor.

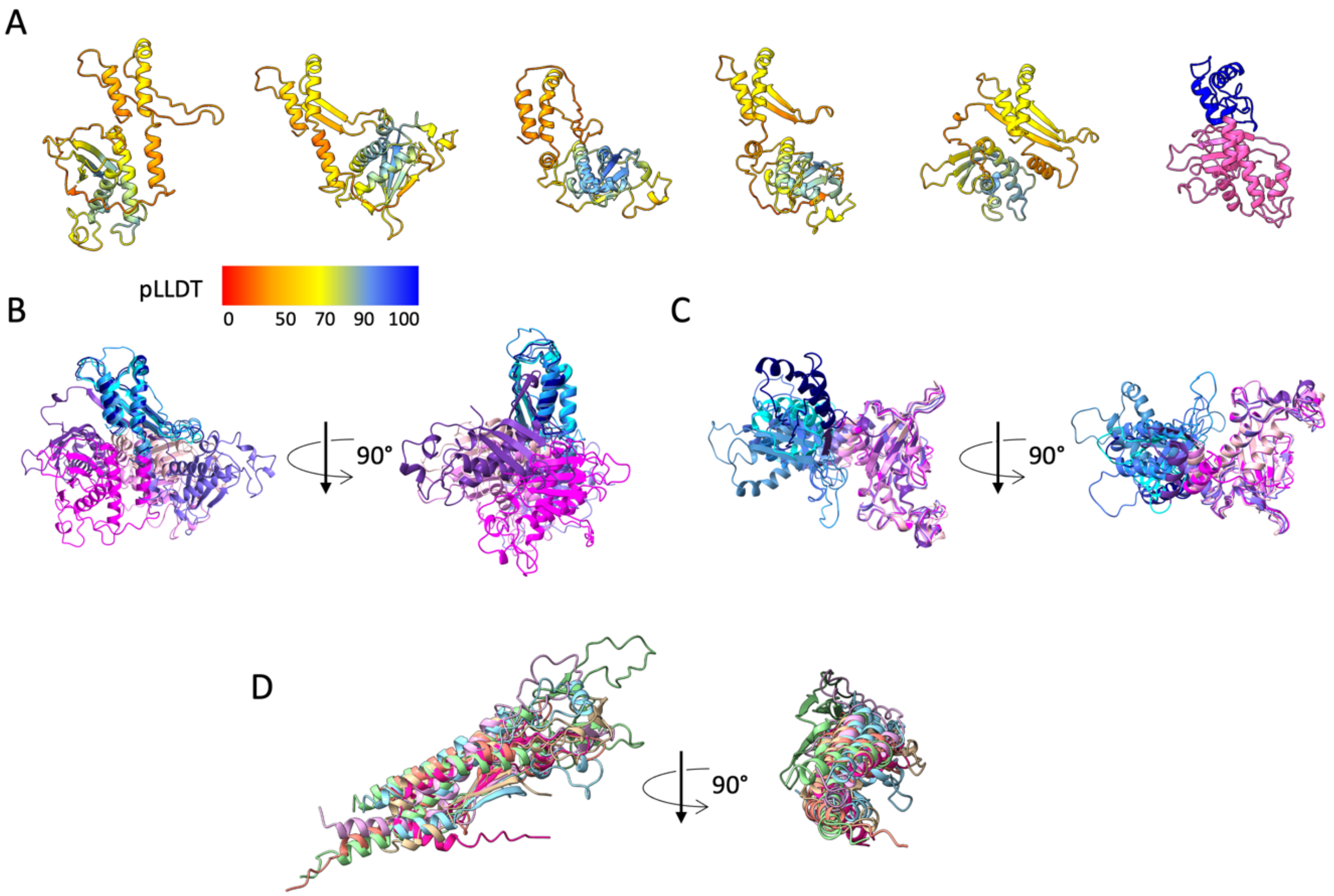

Figure S3: A. Five different AF2 predictions for T5 TCP_{p143}, coloured according to their per-residue pLLDT. The sixth model of the panel is AF2 TCP_{p143} predicted model after flexible fitting in the T5 tip map, with residues 1-80 and 81-255 respectively coloured in blue and pink. **B, C.** Superimposition of the five TCP_{p143} models shown in A, after alignment on residues 1-80 (B, rmsd between 65 pruned atom pairs is 1.057 Å, across all 80 residues: 1.958) or residues 81-255 (C, rmsd between 137 pruned atom residues is 0.669 Å, across all 175 residues: 3.996) using the Matchmaker tool of ChimeraX. Residues 1-80 are coloured in shades of blue and residues 81-255 in shades of pink. **D.** DALI pairwise alignment of AF2 predictions for TCPs from phages λ (green), SPP1 (beige), HK97 (orange), TP901-1 (purple), P2 (pink) and Mu (blue).

Table S1: Table of the relevant DALI hits for T5 Trp_{p142}

PDB code	Z-score	rmsd	lali	nres	%id	Protein name	Structure determination method
8HQO	23.9	1.2	158	160	97	gp119 from siphophage DT57A*	Cryo-EM, in the full phage
6TOA	14	3	128	134	8	Adaptor protein RCC01688 from GTA (empty)	Cryo-EM, in the full particle
6TE9	14	3	131	134	8	Adaptor protein RCC01688 from GTA (native)	Cryo-EM, in the full particle
2GJV	13	3	128	136	9	Putative cytoplasmic protein STM4215 (Salmonella prosiphophage)	X-ray diffraction, crystallised as a hexamer
4ACV	13	2	115	119	7	"Antigen B", prophage λ LM01	X-ray diffraction, crystallised as a hexamer
6U5G	13	3	130	164	8	Collar protein PA0615 from Pyocin R2	Cryo-EM, in the full particle
3FZ2	13	3	128	131	16	TrP gpU from siphophage λ	X-ray diffraction, crystallised as a hexamer
8K37	12.3	2.7	125	131	18	TrP gpU from siphophage λ	Cryo-EM, in the full phage
7KJK	10	3	120	159	10	Tail terminator gp5 from XM1	Cryo-EM, in the full phage
6J0F	10	4	127	272	9	Pvc16 cap protein from PVC (extended state)	Cryo-EM, in the full particle
3J2N	9,7	4	131	211	11	Tail connector protein gp15 from myophage T4 (contracted state)	Cryo-EM (25 Å resolution) + X-ray diffraction structure (PDB: 4HUD)
3J2M	9,3	4	136	211	11	Tail connector protein gp15 from myophage T4 (extended state)	Cryo-EM (25 Å resolution) + X-ray diffraction structure (PDB: 4HUD)
4HUD	9,4	4	131	211	11	Tail connector protein gp15 from myophage T4	X-ray diffraction, crystallised as a hexamer
2L25	9,2	4	129	141	12	Bordetella Bronchisepta Phage Related Protein	NMR, soluble monomer
6RAP	9	3	122	275	8	AFP1 protein from AFP *Serratia entomophila*	Cryo-EM, in the full particle
2LFP	8,7	3	114	139	11	Tail-to-head joining protein gp17 from siphophage SPP1	Cryo-EM (7-8 Å resolution, PDB 5A20 & 5A21) + NMR structure of the soluble monomer
1Z1Z	7,6	3	108	129	11	TrP protein gpU from siphophage λ	NMR, soluble monomer
5N7L	5,4	3	76	80	11	Protein L (GspL) from Type II Secretion System	X-ray diffraction
2RJZ	6,3	3	85	130	7	Type 4 fimbrial biogenesis protein, *P. aeruginosa*	X-ray diffraction
8HDR						Trp gp12 from myophage Pam3	Cryo-EM, in the full phage
8GTF						Trp vBDshSR4C_010 from myophage Dinoroseobacter vB_DshS-R4C	Cryo-EM, in the full phage
8FVH						Gateway protein gp29 from Pseudomonas phage E217	Cryo-EM, in the full phage

*Protein gp119 from DT57C in Ayala *et al.* (22) is called TCP and is wrongly indicated as TCP ORF T5.147 homologue. It is homologous to TrP ORF T5.146, which codes for TrP_{p142}.

Table S2: Validation statistics and model building

	Proximal extremity of T5 tail (EMD-15967)(PDB 8BCP)	Proximal extremity of T5 tail after interaction with FhuA (EMD-15968) (PDB 8BCU)
**Data collection and processing**		
Magnification	105.000 x	105.000 x
Voltage (kV)	300	300
Electron exposure (e^{-}/Å^2)	40	40
Defocus range (μm)	-1.0 to -3.0	-1.0 to -3.0
Pixel size (Å)	1.351	1.351
Symmetry imposed	C3	C3
Micrographs (no.)	3208	5752
Final particle images (no.)	9953	10701
Map resolution (Å) 0.143 FSC threshold	3.88	4.05
Map resolution range (Å)	3.7 – 7	4 – 7
**Refinement**		
Model resolution (Å) 0.5 FSC threshold	4.1	4.1
Map sharpening *B* factor (Å^2)	-146	-120
Model composition		
Chain count	9	9
Non-hydrogen atoms	18171	18279
Protein residues	2337	2349
Ligands	0	0
B factors (Å^2)		
Protein (min/max/mean)	43.48/297.70/89.1	65.51/243.36/101.07
R.m.s. deviations		
Bond lengths (Å)	0.006	0.005
Bond angles (°)	1.005	0.971
Validation		
MolProbity score	1.97	2.04
Clashscore	10.41	12.32
Poor rotamers (%)	0	0.15
Ramachandran plot		
Favoured (%)	96.51	93.26
Allowed (%)	3.49	6.61
Disallowed (%)	0	0.13
